# Infrared Perfect Ultra-narrow Band Absorber as Plasmonic Sensor

**DOI:** 10.1186/s11671-016-1705-1

**Published:** 2016-11-02

**Authors:** Dong Wu, Yumin Liu, Ruifang Li, Lei Chen, Rui Ma, Chang Liu, Han Ye

**Affiliations:** State Key Laboratory of Information Photonics and Optical Communications, Beijing University of Posts and Telecommunications, Beijing, 100876 China

**Keywords:** Plasmonics, Optical sensing and sensors, Metamaterials, Absorption

## Abstract

We propose and numerically investigate a novel perfect ultra-narrow band absorber based on a metal-dielectric-metal-dielectric-metal periodic structure working at near-infrared region, which consists of a dielectric layer sandwiched by a metallic nanobar array and a thin gold film over a dielectric layer supported by a metallic film. The absorption efficiency and ultra-narrow band of the absorber are about 98 % and 0.5 nm, respectively. The high absorption is contributed to localized surface plasmon resonance, which can be influenced by the structure parameters and the refractive index of dielectric layer. Importantly, the ultra-narrow band absorber shows an excellent sensing performance with a high sensitivity of 2400 nm/RIU and an ultra-high figure of merit of 4800. The FOM of refractive index sensor is significantly improved, compared with any previously reported plasmonic sensor. The influences of structure parameters on the sensing performance are also investigated, which will have a great guiding role to design high-performance refractive index sensors. The designed structure has huge potential in sensing application.

## Background

In recent years, plasmonic metamaterials have attracted increasing interest owing to their potential applications in high sensitive photodetection [1], hot electron collection [2, 3], and biosensing [4–8]. Perfect narrow band absorber based on plasmonic metamaterial is a rapidly developing area of research owing to their various applications in energy harvesting [9–14] and thermal emitters [15–18]. The localized surface plasmon resonance (LSPR) is attributed to collective behavior of electrons as the incident wave interacts with metallic nanostructures. Due to the excellent characters of LSPR in confining light at the nanoslit and transforming it into thermal energy, metallic metamaterials possess a great advantage to design absorber. So far, various perfect absorbers have been designed and demonstrated over different frequency ranges. Landy designed and demonstrated the first perfect metamaterial absorber consisting of two electric ring resonators [19]. Tao proposed and experimentally demonstrated a terahertz metamaterial absorber working over a wide range of angles of incidence, which consists of two metallic layers separated by a dielectric layer [20]. A perfect absorber is designed by Hedayati in the visible region by a combination of a metal film with suitable metal-dielectric nanocomposites [21].

When the plasmonic metamaterial structures are surrounded by gas and liquid, a spectral shift of the resonance wavelength can be occurred due to the change of refractive index of environment. Thus, in practical application, narrow band absorbers are often used as biosensor, owing to the narrower band to improve the sensing performance. In designing sensors, the wavelength sensitivity (*S*) and the FOM are generally used for evaluating their performance, where the sensitivity and figure of merit are defined as *S* = *Δλ*/*Δn*, FOM = *S*/FWHM respectively. The Δλ is the resonance wavelength change of reflectance spectrum, which results from the refractive index change of surrounding environment, and FWHM is the full width at half maximum of the reflectance spectrum. As we all know, the higher FOM of refractive index sensor means the bio-sensor with better performance of molecule detection. Thus, it is very meaningful to design an ultra-high FOM refractive index sensor with a simple structure. Unfortunately, the previously reported plasmonic sensors based on metamaterial structure generally have a relatively low FOM <600 [22–39], which will severely limit their further development and application. Shen designed a gold mushroom array structure with a narrow FWHM of 10 nm and a high FOM of 108 [22]. Liu designed a cross-shape patch array structure with FWHM of 12 nm and *S* of 538 nm/RIU [23]. Lin proposed and analyzed based on bowtie nanoantenna arrays (BNAs) with a FOM of 254 [24]. Lu proposed a nanolit microcavity-based structure and demonstrated a narrower FWHM of 8 nm and a FOM of 25 [25]. Li designed and investigated a plasmonic sensor with a FOM of 120 based triple-band metamaterial [26]. Recently, Srekanth proposed a plasmonic refractive index sensor based on a hyperbolic metamaterial with a FOM of 590 [27]. So, the plasmonic sensors based on metamaterial structure in most previous studies are either complicated or have lower FOM.

In this paper, we demonstrate a novel and easily fabricated plasmonic ultra-narrow band absorber based on a two-dimensional metal-dielectric-metal-dielectric-metal (MDMDM) periodic structure, consisting of gold nanobar array and a gold thin film separated by a dielectric layer operating at the near-infrared region. As a plasmonic refractive index sensor, the structure has a high wavelength sensitivity of 2400 nm/RIU as well as an ultra-narrow absorption bandwidth (FWHM) of 0.5 nm. Thus, the FOM of the proposed plasmonic sensor can reach 4800. As far as we know, this is the highest FOM compared with previously reported plasmonic refractive index sensor [22–39]. In order to evaluate the sensing performance of the plasmonic structure, we also investigate the sensitivity of the plasmonic sensor dependence on different structure parameters. By adjusting the structure parameters, the optimized absorption peak or FWHM can be achieved. Moreover, compared with previously reported plasmonic sensor, the metamaterial is simple in structure and easy to manufacture. Importantly, owing to the ultra-high FOM, the LSPR-based sensor possesses huge potential in biomedical and chemical fields.

## Methods

**Fig. 1:**
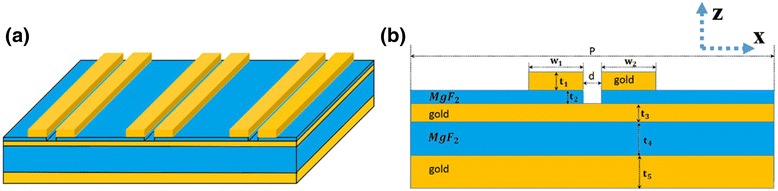
**a** Schematic of one unit cell in the proposed structure. **b** The cross section of the designed structure parameters

Figure 1a illustrates the designed geometry of the metamaterial structure, which consists of gold nanobar periodic array on a thin gold film separated by a dielectric layer. The cross section of the designed structure parameters are shown in Fig. 1b. The gap *d* between two nanobars in one unit cell is d = 20 nm. And other structure parameters include top layer nanobar width *w*_1_ = *w*_2_ = *w*, nanobar thickness *t*_1_, dielectric layer thickness *t*_2_, gold film thickness *t*_3_, and period *p*. In the infrared region, permittivity of gold can be reasonably characterized by the Drude model. The refractive index of the MgF_2_ layer is set as 1.37. The proposed structure is investigated by changing the surrounding refractive index and measuring the absorption spectra.

To investigate the sensing performance of the designed structure, we apply two-dimensional finite-difference time-domain (FDTD) simulation in calculations. In our simulation, we set period boundary conditions in the *x* direction. The optimized geometric parameters of the sensor are set as follows: *w* = 360 nm, *t*_1_ = 20 nm, *t*_2_ = 10 nm, *t*_3_ = 25 nm, *t*_4_ = 170 nm, *t*_5_ = 100 nm, and *p* = 2400 nm. A plane wave is normally incident onto the sensor along the -*z* direction, with its electric field *E* along the *x* direction. Because the thickness *t*_5_ of the gold film is thick enough to forbid the transmission of the incident light (*T* = 0), the absorption could be simplified to be *A* = 1 − *R*.

## Results and Discussion

**Fig. 2:**
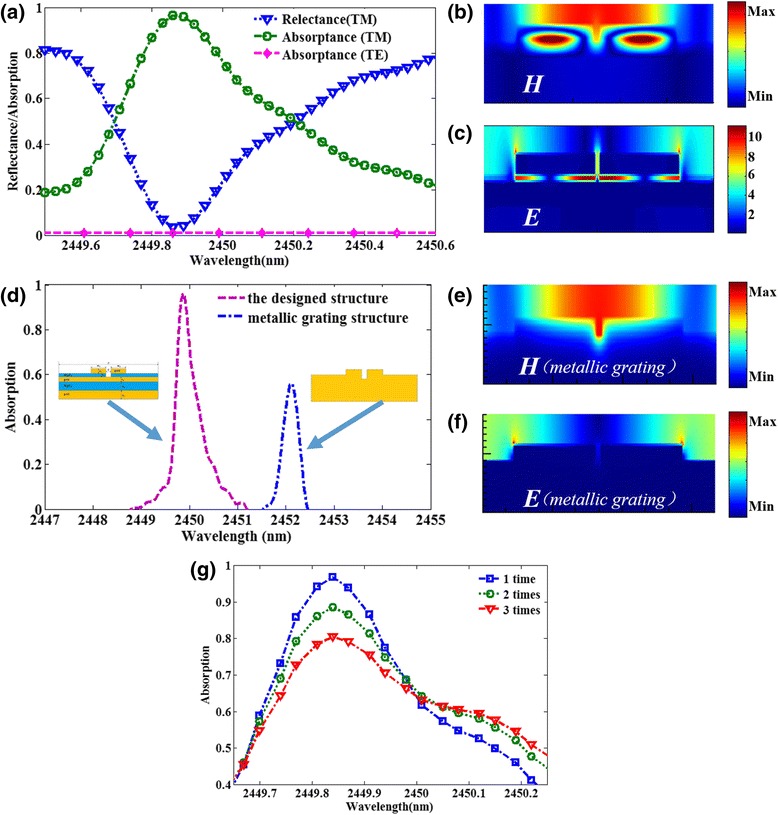
**a** The reflection and absorption spectra of the proposed structure under different polarization configurations. **b** The magnetic field distribution and **c** the electric field distribution at the resonant wavelength of the structure. **d** The comparison chart of absorption spectrum between the designed structure and metallic grating structure. **e** The magnetic field distribution and **f** the electric field distribution at the resonant wavelength of the metallic grating structure. **g** Simulated absorbance spectra when the damping constant of the gold film is two and three times that of bulk gold

The characteristics of the simulated absorption and reflection spectra of the designed structure are very important to evaluate the sensor performance. The absorption spectra at normal incidence for different polarization configurations are studied and shown in Fig. 2a. It is easy to observe that there exists an absorption peak for the TM polarization and no absorption occurs for the TE polarization. This feature can be well explained by the asymmetrical structure of the metamaterial. As shown in Fig. 2a, for TM polarization configuration, when the refractive index of the sensing material is 1.02, the resonance absorption peak of the structure is found at 2449.87 nm with FWHM of 0.5 nm, which is much narrower than previously reported plasmonic refractive index sensor [22–39]. The magnetic field *H* and electric field *E* distributions at resonance are calculated and depicted in Fig. 2b, c, respectively. Figure 2b illustrates that the magnetic field mainly locates in the dielectric spacer among two gold nanobars and the thin gold film, which indicates the coupling effect of the nanostructures caused by LSPR. To better interpret the physical mechanism of the plasmonic absorber, the absorption spectrum is compared between the designed structure and metallic grating structure (see insert of Fig. 2d) in Fig. 2d. The absorption peak of the designed structure is obviously higher than that of the metallic grating structure. The magnetic field *H* and electric field *E* distributions of the metallic grating structure are presented in Fig. 2e, f, respectively. As shown in Fig. 2e, the magnetic field is concentrated in the surface of the gold nanobars. Then, compared with the magnetic field of the designed structure coupled into the dielectric layer in Fig. 2b, the metallic grating structure will theoretically have a poor performance in absorbing ability, which is consistent with the calculated results in Fig. 2d. Therefore, we attribute the ultra-narrow band absorption to the excitation of LSPR between each element in the designed structure. In addition, due to the grain boundary effects and the surface scattering in real thin films, the damping constant of the gold film is likely higher than that of bulk gold [23, 40]. To take this effect into consideration, we also calculate the absorption spectra for damping constant of two and three times that of bulk gold. As shown in Fig. 2g, absorbance peaks with different amplitude and FWHM are observed. The material loss would deteriorate the performance of the designed narrowband absorber [23, 40]. The coupling behavior in the metamaterial structure also can result in the enhancement of electric field intensity. Figure 2c shows that nearly all the electric field is confined to the nanoslits between gold nanobars and the thin gold film and the electric field intensity in extremely tiny volume is about 11 times larger than the incident waves, which have great potential applications in hot electron generation and biosensor.

**Fig. 3:**
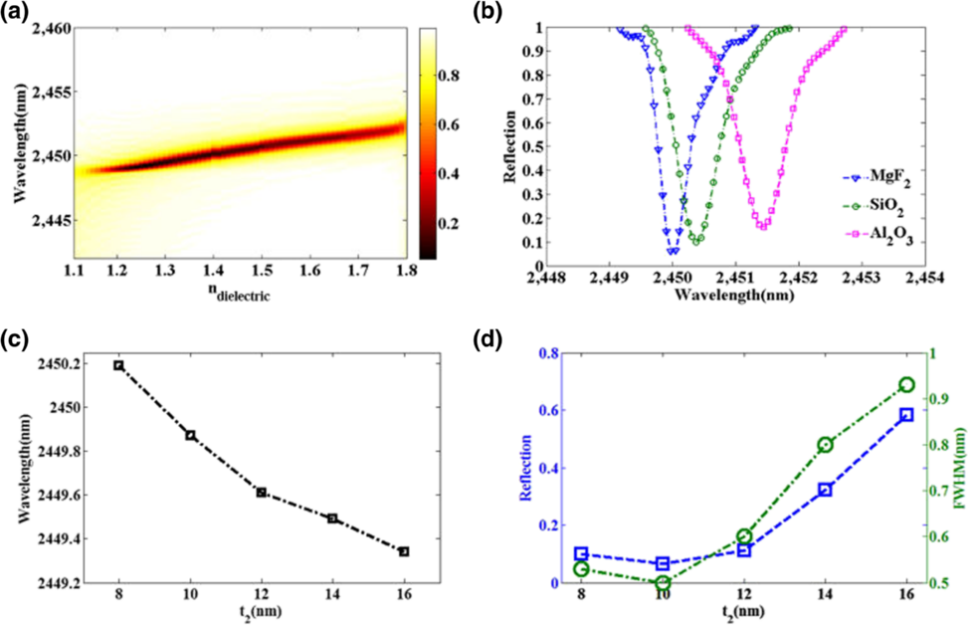
**a**, **b** The dependence of reflective spectra of the designed structure on the index of dielectric. **c** The resonance wavelength as a function of dielectric spacer thickness *t*_2_. **d** Reflectivity of the resonance dip and FWHM as functions of dielectric spacer thickness *t*_2_

As shown in Fig. 3, the influences of materials in dielectric layer on the reflection spectrum are investigated. Figure 3a shows that, when the refractive index of dielectric is increased from 1.1 to 1.8, the resonant wavelength of reflection spectrum redshifts slightly. We provide a comparative analysis of the reflection spectrum using three common dielectric materials (*MgF*_2_, *SiO*_2_, *Al*_2_ *O*_3_) as shown in Fig. 3b. The plasmonic sensor using *MgF*_2_ can achieve the better sensing performance than sensors used *SiO*_2_ and *Al*_2_ *O*_3_, due to the narrower FWHM and lower reflectivity dip. Figure 3c shows a blueshift of resonant wavelength with the thickness *t*_2_ of the dielectric spacer increased. At the same time, the reflection dip and FWHM decrease with decreasing the thickness *t*_2_ shown in Fig. 3d. This feature can be explained that the LSPR is enhanced with the decrease of distance between gold nanobars and gold film. The dielectric spacer with a thickness about 10 nm can be manufactured with standard fabrication techniques [41]. The FWHM and reflectivity dip of the reflection curve depend strongly on the coupling strength between the nanobars and the gold film. Thus, the sensing performances are different with various dielectric materials and thickness of dielectric spacer.

**Fig. 4:**
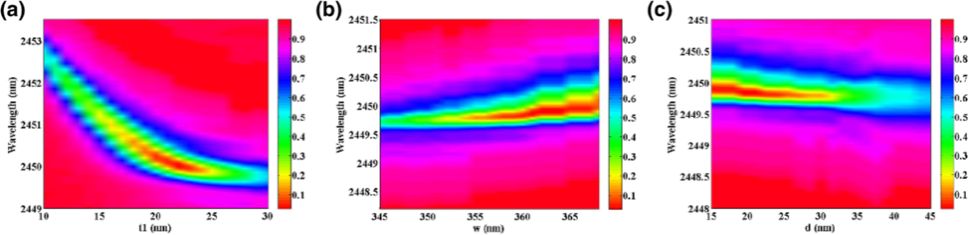
The reflectance spectrum as a function of top layer nanobar **a** thickness *t*_1_, **b** width *w*, and **c** the distance between two top layer nanoribbons *d*, respectively. The refractive index of the surrounding environment is set as 1.02

**Fig. 5:**
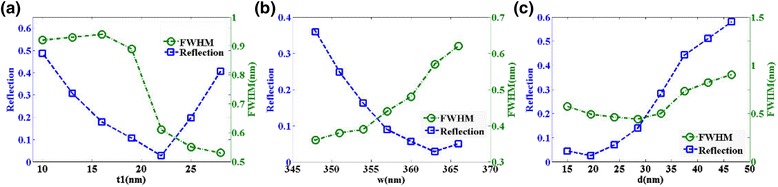
Reflectivity of the resonance dip and FWHM as functions of top layer nanobar **a** thickness *t*_1_, **b** width *w*, and **c** the distance between two top layer nanoribbons *d*, respectively. The refractive index of the surrounding environment is set as 1.02

Figure 4 shows influence of structure parameters on the reflectance spectrum of the proposed metamaterial structure. According to Fig. 4a, when the nanobar thickness *t*_1_ varies from 10 to 30 nm, the resonant wavelength blueshifts obviously. The resonant wavelength of reflectance spectrum redshifts slightly as the nanobar width *w* increases from 340 to 370 nm depicted in Fig. 4b. Figure 4c presents a blueshift of resonant wavelength with the distance between two nanobars *d* increased from 15 to 50 nm. Figure 5 presents the effects of structure parameters on the reflectivity dip and FWHM. Figure 5a shows that the reflectivity of the resonance dip decreases first and then increases obviously with the increase of the thickness of gold nanobar, and the value of FWHM remains at a certain level first and then decreases as the thickness *t*_1_ increases from 10 to 30 nm. As shown in Fig. 5b, when the nanobar width *w* is 363 nm, the reflectivity dip is minimum, and the minimum value of FWHM can reach up to 0.36 nm when the nanobar width *w* is 348 nm, which is far narrower than any previously reported plasmonic sensor. In Fig. 5c, it is easy to observe that the reflectivity is strongly dependent on the distance between two nanobars and the reflectivity of the resonance dip increases obviously when the distance d changes from 15 to 50 nm. This characteristic can be attributed to the reduction of coupling effect between two nanobars with the increase of *d* and then the absorption is weakened. FWHM changes slightly when the distance *d* increases. In practical application, it is generally known that lower reflectivity and narrower FWHM of reflection spectrum is required to enhance the performance of refractive index sensor. From Fig. 5, the optimal value of FWHM and reflectivity cannot be simultaneously obtained. However, in our design, the FWHM changes slightly and the reflectivity of the resonance dip remains low in a wide range, which is favorable to practical application owing to its outstanding robustness.

**Fig. 6:**
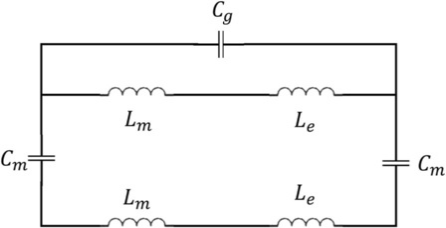
Schematic of the equivalent LC circuit for the structure shown in Fig. 1

As shown in Fig. 2b, the high absorption is caused by the magnetic resonance resulting from LSPR. The equivalent LC circuit model can be used to explain the characteristics of the reflection resonant dip in this work [42–46]. Here, the mutual inductance *L*_*m*_ of the gold nanobars and gold film can be represented by *L*_*m*_ = 0.5 μ_0_ *wt*_2_, where μ_0_ is the permeability of surrounding environment [44–46]. Owing to the contribution of the drifting electrons to the inductance, the kinetic inductance *L*_*e*_ is given by ${L}_e=w/\left(\gamma {\varepsilon}_0{t}_1{\omega}_p^2\right)$, where *γ* is a factor considering the effective cross-sectional area of the gold nanobars, *ε*_0_ is the dielectric permittivity of surrounding environment and *ω*_p_ is the plasma frequency of the gold [44–46]. On the other hand, the gap capacitance ${C}_g=\pi {\varepsilon}_0/{ \ln}^{\left(d/{t}_1\right)}$ is used to represent the capacitance between the two nanobars. The parallel-plate capacitor *C*_*m*_ between the upper gold nanobars and the gold film is expressed as *C*_*m*_ = *c*_1_ *ε*_2_ *ε*_0_ *w*/*t*_2_, where *c*_1_ is a numerical factor accounting for the non-uniform charge distribution at the metal surfaces and *ε*_2_ is the dielectric permittivity of dielectric spacer. According to the equivalent circuit model in Fig. 6, the total impedance is expressed as [44–46]

1 $$ {Z}_{\mathrm{tot}}=\frac{i\omega \left({L}_m+{L}_e\right)}{1-{\omega}^2{C}_g\left({L}_m+{L}_e\right)}-\frac{2i}{\omega {C}_m}+i\omega \left({L}_m+{L}_e\right) $$

Then, the resonance wavelength can be obtained by zeroing the impedance. From the magnetic field distribution shown in Fig. 2b, the coupling between the two gold nanobars is much weaker than that between gold nanobars and the gold film, due to the large gap between the nanobars. When *C*_*g*_ is less than 5 % of *C*_*m*_, the effect of *C*_*g*_ can be neglected. Then, the resonance wavelength of the structure can be obtained by [45]

2 $$ {\lambda}_r\approx 2\pi {c}_0\sqrt{\left({L}_m+{L}_e\right){C}_m} $$

where ${L}_m=0.5\kern0.5em {\upmu}_0{\mathrm{wt}}_2,\kern0.5em {L}_e=w/\left(\gamma {\varepsilon}_0{t}_1{\omega}_p^2\right)$ and *C*_*m*_ = *c*_1_ *ε*_2_ *ε*_0_ *w*/*t*_2_. The relationship between resonance wavelength and structural parameters (nanobar width *w*, nanobar thickness *t*_1_, dielectric spacer thickness *t*_2_) can be predicted approximately by Eq. (2). In the LC model, obviously, the resonance wavelength *λ*_*r*_ increases with increasing the *w* and *ε*_2_. The larger *t*_2_ will cause smaller values for *L*_*e*_ *C*_*m*_, while the other term *L*_*m*_ *C*_*m*_ is independent on the *t*_2_. Similarly, larger *t*_1_ will lead to smaller *L*_*e*_ *C*_*m*_ and the other term *L*_*m*_ *C*_*m*_ is also independent on the *t*_1_. Thus, the resonance wavelength *λ*_*r*_ decreases with increasing the thickness *t*_1_ and *t*_2_. These predicted resonance wavelengths are in good agreement with the simulated results on the influence of *n*_dielectric_, *t*_2_, *w*, and *t*_1_ shown in Figs. 3a, c and 4a, b. The *d* can only influence the value of ${C}_g=\pi {\varepsilon}_0/{ \ln}^{\left(\mathit{\mathsf{d}}/{\mathit{\mathsf{t}}}_1\right)}$. Owing to the weakness of *C*_*g*_, the effect of *d* on resonance wavelength *λ*_*r*_ may be extremely slight, which matches the simulated results shown in Fig. 4d quite well.

**Fig. 7:**
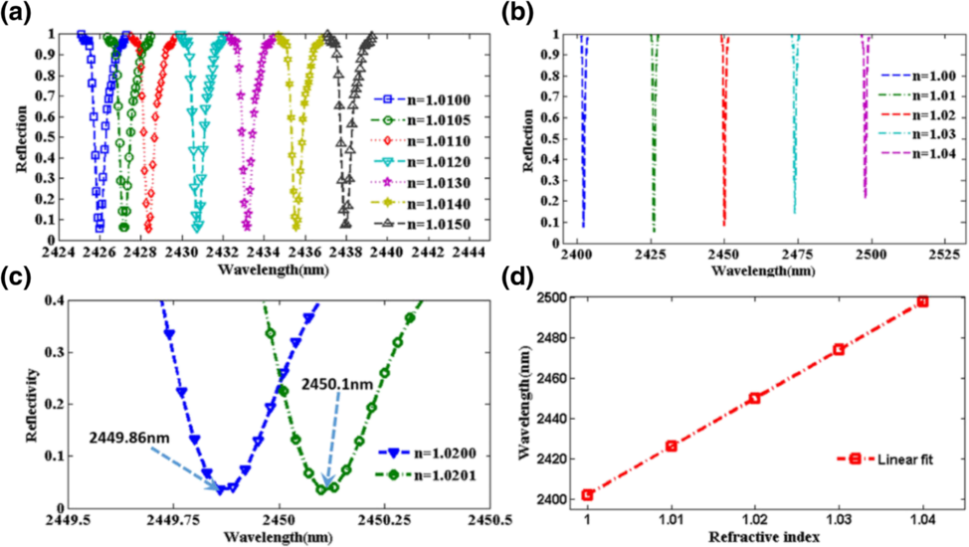
**a**-**c** Simulated reflective spectra of the sensor with different refractive index of environment. **d** Resonant wavelength of the sensor as a function of the surrounding refractive index

As is well known, the resonant wavelength of plasmonic nanostructures is dependent on the refractive index of the surrounding dielectric environment, a property that has been widely utilized for sensing applications. According to Eq. (2), the term *L*_*m*_ *C*_*m*_ will increase with increasing the dielectric permittivity *ε*_0_ of surrounding environment and *L*_*m*_ *C*_*m*_ is independent on the *ε*_0_. Therefore, the resonance of reflection redshifts as the refractive index of surrounding environment increases in the LC model. Then, the sensing characteristics of the designed metamaterial structure are investigated in Fig. 7. According to Fig. 7a, b, the resonance of reflection redshifts as the refractive index of surrounding environment increases. Particularly, in Fig. 7c, the blue to green curves present resonant wavelength of 2449.8 to 2450.1 nm when the surrounding refractive index changes from 1.0200 to 1.0201 with a step of 0.0001. It is easy to observe that this plasmonic sensor can detect a very small change of refractive index of surrounding environment. As shown in Fig. 7d, the sensitivity (*S*) of the sensor is 2400 nm/RIU, while FWHMs can be narrower than 0.5 nm. Therefore, the FOM of the plasmonic sensor can reach 4800, which is improved remarkably compared to any previously reported plasmonic metamaterial structure [20–37].

**Fig. 8:**
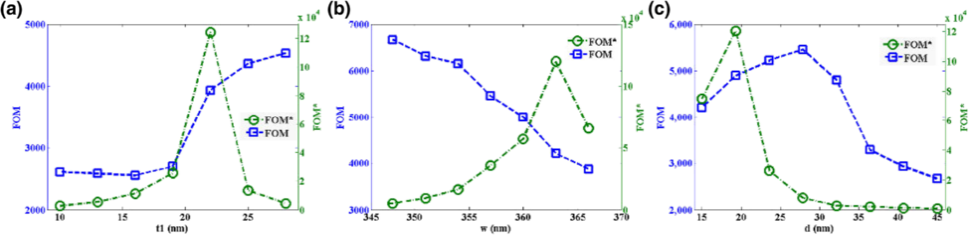
FOM and FOM* as functions of top layer nanobar **a** thickness *t*_1_, **b** width *w*, and **c** the distance between two top layer nanoribbons *d*, respectively

Moreover, the intensity change of reflected wave at a special wavelength can be detected in application and a relative intensity change *dI*/*dn* at the wavelength can be obtained owing to a refractive index change of surrounding environment. Then, the corresponding figure of merit is defined as FOM* = max |(*dI*/*dn*)/*I*|, which can be used for evaluating the ability of detecting the light intensity change of reflected wave, and *I* is the intensity of reflected wave at the fixed wavelength. Then, in order to more clearly describe the sensing performances of the designed metamaterial structure, we calculated the FOM and FOM* from the reflectance spectra, as shown in Fig. 8. Figure 8a shows increase of FOM varying with the thickness *t*_1_ from 345 to 370 nm and a maximum of FOM* = 1.24 × 10^5^ at *t*_1_ = 23 nm. In Fig. 8b, with increasing the *w*, the FOM decreases obviously and has a maximum value 6666.67, which is greater than FOM of any previously reported plasmonic refractive index sensor [20–37]. As shown in Fig. 8c, the FOM and FOM* increase first and then decrease as the *d* changes from 15 to 45 nm. These studies of this ultra-high FOM sensor will have a great guiding role to design high-performance sensors.

## Conclusions

In this paper, using finite-difference time-domain (FDTD) simulation, we propose and numerically investigate a novel ultra-narrow bandwidth plasmonic absorber based on a MDMDM periodic structure at near-infrared wavelengths. The metamaterial absorber shows an ultra-narrow absorption bandwidth (FWHM) of 0.5 nm with absorption peaks over 98 % at normal incidence. The high absorption is ascribed to the coupling effect between gold nanobars and the gold film resulting from the excitation of LSPR. Importantly, this plasmonic structure presents excellent sensing performance with a high wavelength sensitivity of 2400 nm/RIU and an ultra-high FOM of 4800. To the best of our knowledge, this is the highest value of FOM compared with any reported plasmonic sensor to date. Then, we investigate the influence of the structure parameters on the performance of the plasmonic sensor. Moreover, the designed structure also can show the strong electric field confinement and enhancement in a nanogap region. Due to the ultra-high FOM and the high sensitivity, our metamaterial structure achieves a promising way to realize ultra-high resolution refractive index sensor based on LSPR, which has great potential in biomedical and chemical applications.

## Abbreviations

BNAs: Nanoantenna arrays
FDTD: Finite-difference time-domain
FOM: Figure of merit
FWHM: Full width at half maximum
LSPR: Localized surface plasmon resonance
MDMDM: Metal-dielectric-metal-dielectric-metal
